# Association between statin therapy and outcomes in critically ill patients: a nested cohort study

**DOI:** 10.1186/1472-6904-11-12

**Published:** 2011-08-06

**Authors:** Shmeylan A Al Harbi, Hani M Tamim, Yaseen M Arabi

**Affiliations:** 1 Intensive Care Department, King Abdulaziz Medical City, Riyadh, Saudi Arabia; 2 Epidemiology and Biostatistics, College of Medicine, King Saud bin Abdulaziz University for Health Sciences, King Abdulaziz Medical City, Riyadh, Saudi Arabia; 3 Intensive Care Department, Medical Director, Respiratory Services, and Associate Professor, College of Medicine, King Saud bin Abdulaziz University for Health Sciences, King Abdulaziz Medical City, Riyadh, Saudi Arabia

## Abstract

**Background:**

The effect of statin therapy on mortality in critically ill patients is controversial, with some studies suggesting a benefit and others suggesting no benefit or even potential harm. The objective of this study was to evaluate the association between statin therapy during intensive care unit (ICU) admission and all-cause mortality in critically ill patients.

**Methods:**

This was a nested cohort study within two randomised controlled trials conducted in a tertiary care ICU. All 763 patients who participated in the two trials were included in this study. Of these, 107 patients (14%) received statins during their ICU stay. The primary endpoint was all-cause ICU and hospital mortality. Secondary endpoints included the development of sepsis and severe sepsis during the ICU stay, the ICU length of stay, the hospital length of stay, and the duration of mechanical ventilation. Multivariate logistic regression was used to adjust for clinically and statistically relevant variables.

**Results:**

Statin therapy was associated with a reduction in hospital mortality (adjusted odds ratio [aOR] = 0.60, 95% confidence interval [CI] 0.36-0.99). Statin therapy was associated with lower hospital mortality in the following groups: patients >58 years of age (aOR = 0.58, 95% CI 0.35-0.97), those with an acute physiology and chronic health evaluation (APACHE II) score >22 (aOR = 0.54, 95% CI 0.31-0.96), diabetic patients (aOR = 0.52, 95% CI 0.30-0.90), patients on vasopressor therapy (aOR = 0.53, 95% CI 0.29-0.97), those admitted with severe sepsis (aOR = 0.22, 95% CI 0.07-0.66), patients with creatinine ≤100 μmol/L (aOR = 0.14, 95% CI 0.04-0.51), and patients with GCS ≤9 (aOR = 0.34, 95% CI 0.17-0.71). When stratified by statin dose, the mortality reduction was mainly observed with statin equipotent doses ≥40 mg of simvastatin (aOR = 0.53, 95% CI 0.28-1.00). Mortality reduction was observed with simvastatin (aOR = 0.37, 95% CI 0.17-0.81) but not with atorvastatin (aOR = 0.80, 95% CI 0.84-1.46). Statin therapy was not associated with a difference in any of the secondary outcomes.

**Conclusion:**

Statin therapy during ICU stay was associated with a reduction in all-cause hospital mortality. This association was especially noted in high-risk subgroups. This potential benefit needs to be validated in a randomised, controlled trial.

## Background

Statins, also known as 3-hydroxy-3-methylglutaryl coenzyme A reductase inhibitors, were first introduced in the late 1980s as cholesterol-lowering drugs for the prevention of cardiovascular events. However, recent studies have demonstrated a wide variety of statin properties independent of their lipid-lowering ability. These properties, known as pleiotropic effects [1-4], include multiple anti-inflammatory actions, the direct activation of heme oxygenase, direct interference in leucocyte-endothelial interactions, and direct inhibition of major histocompatibility complex class II (MHC II) [5-10].

The effect of statin therapy on mortality in critically ill patients is controversial, with some studies suggesting a benefit and others no benefit or even potential harm [11-17]. These divergent results are probably related to differences in study designs, patient populations [4,11-14,18,19], statin types, and doses [4,15,17,20]. Therefore, we sought to evaluate the association of statin therapy during the intensive care unit (ICU) stay with all-cause mortality in critically ill medical surgical patients.

## Methods

### Setting

This study was conducted in a 900-bed tertiary academic medical centre. The adult ICU admits medical, surgical, and trauma patients, and it operates as a closed unit with 24-hr, 7-day onsite coverage by a critical care board of certified intensivists. The nurse-to-patient ratio at the unit is approximately 1:1.2 [21].

### Study design

This was a nested cohort study within two randomised, controlled trials.

The first trial, conducted between January 2004 and March 2006 included 532 patients, compared intensive insulin therapy (IIT) (for patients with a blood glucose level of 4.4-6.1 mmol/L or 80-110 mg/dl) to conventional insulin therapy (CIT) (for patients with a blood glucose level of 10-11.1 mmol/L or 180-200 mg/dl) [22]. This trial showed no significant difference in ICU mortality between the IIT and CIT groups (13.5% vs. 17.1%, p = 0.3). Hypoglycemia occurred more frequently in the IIT than in the CIT group (28.6% vs. 3.1% of patients; p < 0.0001) [22].

The second trial, conducted between February 2006 and January 2008, included 240 patients and assessed the effects on outcomes of permissive underfeeding (a caloric goal of 60-70% of the calculated requirement) versus target feeding (caloric goal of 90-100% of the calculated requirement) with either IIT or CIT in critically ill patients [23]. The study found no difference between the two groups in 28-day mortality (18% vs. 23%, p = 0.34). However, hospital mortality was lower in the permissive underfeeding compared with the target-feeding group (30% vs. 43%, p = 0.04) [23]. All patients enrolled in the original two trials were included in this study.

### Statin therapy

Statins for critically ill patients were prescribed as part of the medication reconciliation process if they had been prescribed in the pre-ICU period. Occasionally, statin therapy was initiated in the ICU for patients admitted with acute coronary syndrome or stroke. The prescribed dose was at the discretion of the treating physician. Data regarding the use, type (simvastatin or atorvastatin) and dose of statin were collected from the hospital information system. Doses of atorvastatin were converted into the equivalent dose of simvastatin at a atorvastatin: simvastatin ratio of 1:2.

### Data Collection

Patient outcomes and the following data were retrieved from the two original studies: age, gender, acute physiology and chronic health evaluation (APACHE II) score [24], sequential organ failure assessment (SOFA) score [25], creatinine, platelet count, bilirubin, international normalised ratio (INR), Glasgow coma scale (GCS) score [26], admission category, history of diabetes, need for mechanical ventilation, vasopressor therapy, sepsis, severe sepsis and septic shock and the presence of chronic cardiac, respiratory, renal, hepatic, or immunocompromising diseases, as defined by the APACHE II system [24].

### Outcomes

The primary outcomes were all-cause ICU and hospital mortality. Secondary outcomes included the development of sepsis and severe sepsis during the ICU stay, ICU and hospital length of stay, and the duration of mechanical ventilation. Sepsis and severe sepsis were defined according to the 2001 International Sepsis Definitions Conference [27]. The two trials were approved by the research committee and institutional review board of King Abdulaziz Medical City.

### Statistical analysis

Statistical analyses were performed using Statistical Analysis Software (SAS, release 8, SAS Institute, Cary, NC, 1999). We compared patients who received statins during their ICU admission (statin group) with those who did not (non-statin group). Baseline characteristics and outcome variables were compared using *t*-test for continuous data and Chi-square test for nominal data. To control for any potential confounding effects of baseline characteristics, we used multivariate logistic regression to calculate adjusted odds ratios (aOR) and 95% confidence intervals (CI) for the association between statin use and outcome. Adjustments were made for clinically relevant variables and for those that showed a statistically significant difference between the two groups at baseline. These variables included age, gender, admission category, APACHE II score, history of diabetes, creatinine, platelets, GCS, and the presence of chronic cardiac, renal, or respiratory diseases. We further examined the dose effects of statins on the primary and secondary outcomes by stratifying patients on the basis of doses equivalent to <40 mg and ≥ 40 mg of simvastatin. Additionally, we conducted stratified analyses by age, gender, admission category, APACHE II score, history of diabetes, the presence of chronic cardiac problems, vasopressor therapy, sepsis, severe sepsis and septic shock, creatinine, platelet count, bilirubin, INR, GCS, ICU length of stay, mechanical ventilation and type of statin, to detect any change in the association between intervention and outcome measures on the basis of any of these factors. Statistical significance was defined as a p value ≤ 0.05.

## Results

Of the 763 patients enrolled in the study, 107 (14%) received statins during their ICU stay, and 656 (86%) did not. Atorvastatin was prescribed to 63 patients (58.9%), with doses ranging between 10 and 80 mg/day. Simvastatin was prescribed to 44 patients (41.1%), with doses ranging between 10 and 40 mg/day. Table 1 compares the baseline characteristics of the statin and non-statin groups. Patients who received statins were older; more likely to be females; diabetic; more likely to have chronic cardiac, renal or respiratory illness; and had higher APACHE II scores.

**Table 1:** Baseline characteristics of patients on statin therapy and non-statin therapy

	Statin (n = 107)	Non-Statin (n = 656)	*P*-value
Age, mean ± SD (yrs)	68.8 ± 11.0	49.3 ± 21.7	< 0.0001
Female gender, no. (%)	44 (41.1)	164 (25)	0.0005
APACHE II, mean ± SD	27.2 ± 6.9	23.0 ± 8.1	< 0.0001
SOFA day 1, mean ± SD	9.3 ± 3.2	9.2 ± 3.5	0.92
Creatinine, mean ± SD, μmol/L*	200.8 ± 152.6	152.3 ± 152.1	0.002
Platelets, mean ± SD, x10^9^/L	270.7 ± 117.8	193.3 ± 123.3	0.0001
Bilirubin, mean ± SD, μmol/l	26.4 ± 63.6	31.6 ± 55.2	0.50
INR, mean ± SD	1.5 ± 0.7	1.5 ± 0.9	0.72
GCS, mean ± SD	9.4 ± 4.5	8.5 ± 4.0	0.05
ICU admission category, no. (%)			
Postoperative	4 (3.7)	119 (18.1)	0.0002
Nonoperative	103 (96.3)	537 (81.9)	
History of diabetes, no. (%)	84 (78.5)	219 (33.4)	< 0.0001
Mechanically ventilated, no. (%)	92 (86.0)	591 (90.1)	0.20
Vasopressors, no. (%)	73 (68.2)	423 (64.5)	0.45
Sepsis, no. (%)	28 (26.2)	166 (25.3)	0.85
Severe sepsis, no. (%)	26 (24.3)	207 (31.5)	0.13
Chronic respiratory, no. (%)	33 (30.8)	124 (18.9)	0.005
Chronic cardiac, no. (%)	54 (50.5)	103 (15.7)	< 0.0001
Chronic renal, no. (%)	29 (27.1)	74 (11.3)	< 0.0001
Chronic liver, no. (%)	4 (3.7)	50 (7.6)	0.15
Chronic immunocompromised, no. (%)	10 (9.4)	56 (8.5)	0.78

SD: standard deviation, APACHE II: Acute physiology and chronic health evaluation II; SOFA: Sequential organ failure assessment; INR: International normalised ratio; GCS: Glasgow coma scale.

*To convert units to mg/dl, divide by 88.4 for creatinine and 17.1 for bilirubin

Table 2 summarises the association between statin therapy and mortality using multivariate analysis adjusting for the selected confounders. Statin therapy was associated with lower all-cause hospital mortality (aOR = 0.60, 95% CI 0.36-0.99). The association between statins and ICU mortality was not statistically significant (aOR = 0.84, 95% CI 0.47-1.51). When stratified by statin dose, a significant reduction in hospital mortality was observed with doses of ≥40 mg (aOR = 0.53, 95% CI 0.28-1.00).

**Table 2:** Association between statin use and primary outcomes compared with the non-statin group using adjusted analyses

	Statin n = 107	Non-statin n = 656	Adjusted odds ratio (aOR)^a^	95% confidence interval	*P*-value
ICU mortality, no. (%)	19/107 (17.8)	108/656 (16.5)	0.84	(0.47 - 1.51)	0.56
Dose < 40 mg	7/47 (14.9)	108/656 (16.5)	0.67	(0.28 - 1.63)	0.43
Dose ≥ 40 mg	12/60 (20.0)	108/656 (16.5)	0.97	(0.48 - 1.99)	0.98
Hospital mortality, no. (%)	42/107 (39.3)	200/656 (30.5)	0.60	(0.36 - 0.99)	0.05
Dose < 40 mg	19/47 (40.4)	200/656 (30.5)	0.69	(0.35 - 1.40)	0.30
Dose ≥ 40 mg	23/60 (38.3)	200/656 (30.5)	0.53	(0.28 - 1.00)	0.05

Variables entered initially in the stepwise regression model including age, gender, admission category, APACHE II score, history of diabetes, creatinine, platelets, GCS, PaO_2_/FiO_2_, and chronic cardiac, renal, and respiratory diseases.

Table 3 shows the association between statin therapy and all-cause hospital mortality stratified by different characteristics using multivariate analysis. Statin therapy was associated with lower hospital mortality in patients older than 58 years (aOR = 0.58, 95% CI 0.35-0.97), those with an APACHE II score >22 (aOR = 0.54, 95% CI 0.31-0.96), diabetic patients (aOR = 0.52, 95% CI 0.30-0.90), patients on vasopressor therapy (aOR = 0.53, 95% CI 0.29-0.97), those with creatinine ≤100 μmol/L (aOR = 0.14, 95% CI 0.04-0.51), patients with severe sepsis (aOR = 0.22, 95% CI 0.07-0.66), and patients with a GCS ≤ 9 (aOR = 0.34, 95% CI 0.17-0.71). When stratified by statin type, statistically significant association with lower mortality was observed by simvastatin (aOR = 0.37, 95% CI 0.17-0.81), but not with atorvastatin.

**Table 3:** Association between statin therapy and hospital mortality, stratified by different relevant characteristics

	Statin n = 107	Non-statin n = 656	Adjusted odds ratio (aOR)^a^	95% confidence interval	*P*-value
Hospital mortality, no. (%)	42/107 (39.3)	200/656 (30.5)	0.60	(0.36 - 0.99)	0.05
Stratified by age, no. (%)					
≤ 58 yrs	6/14 (42.9)	62/374 (16.6)	0.73	(0.19 - 2.81)	0.64
> 58 yrs	36/93 (38.7)	138/282 (49.0)	0.58	(0.35 - 0.97)	0.04
Gender, no. (%)					
Male	21/63 (33.3)	126/492 (25.6)	0.56	(0.29 - 1.08)	0.08
Female	21/44 (47.7)	74/164 (45.0)	0.74	(0.35 - 1.58)	0.43
Admission category, no. (%)					
Postoperative	1/4 (25.0)	19/119 (16.0)	0.75	(0.04 - 14.82)	0.85
Nonoperative	41/103 (40.0)	181/537 (34.0)	0.62	(0.34 - 1.03)	0.06
APACHE II, no. (%)					
APACHE≤ 22	8/33 (24.2)	53/370 (14.3)	0.77	(0.27 - 2.21)	0.63
APACHE>22	34/74 (46.0)	147/286 (51.4)	0.54	(0.31 - 0.96)	0.03
SOFA, no. (%)					
≤ 9	13/58 (22.4)	63/346 (18.2)	0.55	(0.26 - 1.17)	0.12
> 9	29/49 (59.1)	137/310 (44.2)	0.73	(0.36 - 1.48)	0.38
History of diabetes, no. (%)					
Yes	31/84 (37.0)	106/209 (48.4)	0.52	(0.30 - 0.90)	0.02
No	11/23 (47.8)	94/437 (21.5)	1.31	(0.49 - 3.54)	0.59
History of cardiovascular, no. (%)					
Yes	23/54 (42.6)	58/103 (56.3)	0.59	(0.3 - 1.2)	0.13
No	19/53 (35.9)	142/553 (25.7)	0.52	(0.27 - 1)	0.06
Vasopressor therapy, no. (%)					
Yes	31/73 (42.5)	151/423 (35.7)	0.53	(0.29 - 0.97)	0.04
No	23/34 (67.7)	184/233 (79.0)	0.92	(0.37 - 2.25)	0.84
Sepsis, no. (%)					
Yes	12/28 (43.0)	88/166 (53.0)	0.64	(0.26 - 1.58)	0.33
No	30/79 (38.0)	112/490 (23.0)	0.72	(0.41 - 1.28)	0.26
Severe sepsis and septic shock no. (%)					
Yes	14/26 (53.9)	84/207 (40.6)	0.22	(0.07 - 0.66	0.007
No	28/81 (34.6)	116/449 (25.7)	0.78	(0.45 - 1.37)	0.38
Creatinine, no. (%)					
≤ 100 μmol/L	4/26 (15.4)	70/351 (20.0)	0.14	(0.04 - 0.51)	0.002
> 100 μmol/L	38/81 (47.0)	130/305 (43.0)	1.01	(0.58 - 1.77)	0.96
Platelets, no. (%)					
≤ 180	12/25 (48.0)	125/358 (35.0)	0.62	(0.24 - 1.62)	0.33
> 180	30/82 (36.6)	75/298 (25.2)	0.78	(0.43 - 1.42)	0.42
Bilirubin, no. (%)					
≤ 16 μmol/L	10/38 (26.3)	54/212 (25.5)	0.49	(0.21 - 1.20)	0.11
> 16 μmol/L	10/22 (45.5)	73/223 (32.7)	0.67	(0.23 - 1.97)	0.47
INR, no. (%)					
≤1.2	20/59 (34.0)	68/312 (21.8)	0.79	(0.40 - 1.58)	0.51
>1.2	22/48 (45.8)	132/344 (38.4)	0.53	(0.26 - 1.09)	0.09
GCS, no. (%)					
≤ 9	19/50 (38.0)	114/364 (31.3)	0.34	(0.17 - 0.71)	0.004
> 9	23/57 (40.4)	86/292 (29.5)	092	(0.46 - 1.85)	0.81
Length of stay, no. (%)					
≤ 5 days	4/39 (10.3)	32/192 (16.7)	0.40	(0.13 - 1.28)	0.12
> 5 days	38/68 (55.9)	168/464 (36.2)	0.63	(0.34 - 1.19)	0.16
Mechanical ventilation, no. (%)					
Yes	40/92 (43.5)	183/591 (31.0)	0.66	(0.34 - 1.10)	0.11
No	2/15 (13.3)	17/65 (26.2)	0.75	(0.09 - 6.43)	0.79
Simvastatin, no. (%)	12/44 (27.3)	200/656 (30.5)	0.37	(0.17 - 0.81)	0.01
Atorvastatin, no. (%)	30/63 (47.6)	200/656 (30.5)	0.80	(0.84 - 1.46)	0.47

APACHE II: Acute physiology and chronic health evaluation II; SOFA: Sequential organ failure assessment; INR: International normalised ratio; GCS: Glasgow coma scale.

*To convert units to mg/dl, divide by 88.4 for creatinine and 17.1 for bilirubin

There was no significant association between statin use and the development of sepsis or severe sepsis and septic shock, ICU and hospital length of stay, or the duration of mechanical ventilation (Table 4).

**Table 4:** Association between statin use and outcomes stratified by statin dose

	Statin therapy n = 107	Non-statin therapy n = 656	Adjusted odds ratio (aOR)^a^	95% confidence interval	*P*-value
Sepsis, no.%	32/107 (29.9)	280/656 (42.7)	0.84	(0.51 - 1.37)	0.49
Dose < 40 mg	16/47 (34.0)	280/656 (42.7)	1.11	(0.57 - 2.20)	0.74
Dose ≥ 40 mg	16/60 (25.7)	280/656 (42.7)	0.67	(0.36 - 1.30)	0.22
Severe sepsis, no. %	26/107 (24.3)	207/656 (31.6)	0.87	(0.51 - 1.50)	0.60
Dose < 40 mg	11/47 (23.4)	207/656 (31.6)	0.88	(0.42 - 1.90)	0.74
Dose ≥ 40 mg	15/60 (25.0)	207/656 (31.6)	0.85	(0.44 - 1.65)	0.64
ICU LOS, mean ± SD, days	10.2 ± 12.7	11.3 ± 10.6	*	*	0.33
Hospital LOS, mean ± SD, days	67.6 ± 118.1	58.6 ± 81.4	*	*	0.37
MVD, mean ± SD, days	9.0 ± 11.9	10.1 ± 10.3	*	*	0.33

Variables entered initially in the stepwise regression model including age, gender, admission category, APACHE II, history of diabetes, creatinine, platelets, GCS, and chronic cardiac, renal, and respiratory diseases.

## Discussion

Our study demonstrates that statin therapy in critically ill patients is associated with lower hospital mortality. This effect was observed predominantly in elderly patients, diabetics, patients with higher severity of illness, with a low GCS, patients on vasopressors, with severe sepsis, and those on simvastatin.

Several studies have shown favourable effects of statin therapy on outcomes in critically ill patients. Liappies et al. retrospectively reviewed patients with documented bacteraemia and found that statin therapy was associated with a significant reduction in overall hospital mortality and infection rates (11). Almog et al. found that prior statin therapy was associated with a reduction in severe sepsis and the incidence of ICU admission [12]. Kruger et al. studied a cohort of bacteraemia patients and found a significantly lower incidence of mortality and bacteraemia-related mortality with statin therapy [13]. Mortensen et al. found that statin therapy before ICU admission was associated with a decreased 30-day mortality [20]. A recent systematic review examined the effect of statins on mortality in patients with infection and/or sepsis. The review included a total of 20 studies, with 18 cohort studies (12 retrospective and 6 prospective), 1 matched cohort study with 2 case-controlled studies, and 1 randomised, controlled trial. The review demonstrated a protective effect of statin therapy for various infection-related outcomes compared with placebo in patients with sepsis and/or other infections [28]. These studies are in concordance with ours, which demonstrated a significant reduction in mortality with statin therapy in critically ill patients and patients with sepsis, severe sepsis and septic shock. The effect was primarily observed with higher doses of statins.

In contrast, some investigators have not found statin therapy to be beneficial. Fernandez et al. analysed data from 438 patients receiving mechanical ventilation for more than 96 hr and found that hospital mortality was significantly higher with statin therapy (61% vs. 42%), even after adjusting for APACHE II predicted risk (observed/expected ratio 1.53 vs. 1.17). In a population-based study of community-acquired pneumonia, Majumdar et al. found that statin users were less likely to die or to be admitted to the ICU than non-users (50/325 [15%] vs. 574/3090 [19%], respectively; OR 0.80, p = 0.15). However, after a more complete adjustment for confounding factors, the OR changed from potential benefit (0.78, adjusted for age and sex) to potential harm (1.10, fully adjusted, including propensity scores, 95% CI 0.76-1.60). This disagreement with the findings of our study may be explained by differences in illness severity. As subjects of a population-based study, the patients of Majumdar et al. were relatively healthy users [15] compared with our population. Additionally, it remains unclear whether these findings, which were observed in patients with community-acquired pneumonia, are applicable to all critically ill patients. Yang et al. conducted a retrospective study and found no difference in mortality between the two groups, despite the presence in patients in the statin group of less organ dysfunction, lower APACHE II scores, less inotropic support, and less shock [16]. The lack of benefit observed in these studies might be related to differences in the patient disease mix, severity of illness, or statin type/dose.

Our study demonstrated a mortality reduction in older patients and those with more severity of illness. This result confirms what other previous studies have shown. Dobesh et al. evaluated patients over the age of 40 with severe sepsis who were exposed to statins before and/or during hospitalisation. The mean APACHE II score in the statin group was 26. Patients in the statin group showed a lower mortality compared with the no statin group (31.7% vs. 48.4%; p = 0.04), and this effect was more evident in older patients and those with higher APACHE II score [18]. Schmidt et al. evaluated statin therapy in patients with multi-organ dysfunction and a mean APACHE II score ≥ 30. The study demonstrated that 28-day mortality was significantly lower in the statin group compared with the no statin group (33% vs. 53%; p = 0.03).

We observed a mortality reduction with simvastatin but not atorvastatin. A similar finding was noted by Christensen et al. [19]. This result might be related to the higher lipophilicity of simvastatin, which enables it to better penetrate cells [29]. However, large sample size is required to confirm these findings.

The observed beneficial effects of statins on diabetic patients, patients with chronic cardiovascular disease and those with low GCS (a surrogate for neurologic disease) are consistent with its reported benefits in acute myocardial infarction and acute stroke [30,31]. Our results are also consistent with the findings of a population-based study by Hackam et al., who found that statin use in patients with atherosclerosis was associated with a reduced risk of subsequent sepsis [32].

Our findings confirm the need for randomised, controlled trials to verify the relationship between statin therapy and the observed outcomes. Other issues still require resolution, including the dose-effect relationship, whether the mechanism of action is related to the pleiotropic or lipid-lowering effect of statins, and whether the observed effect is a class effect or an individual statin effect [33]. We hope that the ongoing clinical trials in patients with sepsis, septic shock, ventilator-associated pneumonia, and influenza, as well as the trials investigating the prevention of acute lung injury and adult respiratory distress syndrome will help address these questions [34-38].

The findings of our study must be interpreted in the light of its strengths and weaknesses. The strengths include being a nested cohort within randomised controlled trials with prospective data collection. Among the limitations of our study are its monocenter nature, its post-hoc design, and the lack of data on the duration of statin therapy prior to ICU admission, lipid profile, and statin side effects. Because of the observational nature of the study, there were imbalances at baseline between the statin and non-statin groups. However, we have adjusted for these imbalances using multivariate analysis.

## Conclusion

Our study demonstrated that statin therapy during ICU admission was associated with lower hospital mortality, particularly among elderly patients, diabetics, patients with high APACHE II and low GCS, those on vasopressors, and those on simvastatin. In addition, using doses greater than 40 mg per day was associated with lower hospital mortality. Further randomised, controlled trials are needed to confirm these findings.

## Competing interests

The authors declare that they have no competing interests.

## Authors' contributions

SAA: Had full access to all data in the study and takes full responsibility for the data, conception and design, participated in the data acquisition, analysis and interpretation, and drafted the manuscript; HMT: Conception and design, data analysis and interpretation and critical revision of the manuscript; YMA: Conception and design, statistical analysis, critical revision of the manuscript and overall supervision.

All authors have read and approved the final manuscript for publication.

## Pre-publication history

The pre-publication history for this paper can be accessed here:

http://www.biomedcentral.com/1472-6904/11/12/prepub
